# Microbial stress mediated intercellular nanotubes in an anaerobic microbial consortium digesting cellulose

**DOI:** 10.1038/s41598-017-18198-w

**Published:** 2017-12-21

**Authors:** Martina John, Antoine Prandota Trzcinski, Yan Zhou, Wun Jern Ng

**Affiliations:** 1 Advanced Environmental Biotechnology Centre, Nanyang Environment and Water Research Institute, Nanyang Technological University, 1 Cleantech Loop, CleanTech One, #06-08, Singapore, 637141 Singapore; 2 Division of Environmental and Water Resources, School of Civil and Environmental Engineering, Nanyang Technological University, 50 Nanyang Avenue, Singapore, 639798 Singapore

## Abstract

The anaerobic digestion process is a multi - step reaction dependent on concerted activities such as exchange of metabolites among physiologically different microbial communities. This study investigated the impact of iron oxide nanoparticles on the anaerobic sludge microbiota. It was shown there were three distinct microbial phases following addition of the nanoparticles: microbial stress and cell death of approximately one log order of magnitude, followed by microbial rewiring, and recovery. Furthermore, it was noted that cellular stress led to the establishment of intercellular nanotubes within the microbial biomass. Intercellular nanotube - mediated communication among genetically engineered microorganisms and ad hoc assembled co - cultures have been previously reported. This study presents evidence of intercellular nanotube formation within an environmental sample – i.e., anaerobic sludge microbiota subjected to stress. Our observations suggested a mode of microbial communication in the anaerobic digestion process not previously explored and which may have implications on bioreactor design and microbial functions.

## Introduction

Treatment of sludge through anaerobic digestion (AD) has been gaining interest because of the process’s relatively low energy requirement, efficiency, and production of a renewable fuel in comparison to aerobic digestion^1–3^. Despite the popularity of conventional AD, one of the major drawbacks of this technology is the comparatively long sludge retention time, largely attributable to the slow growth rate of the anaerobic methanogens^4^ and to hydrolysis. The latter is frequently identified as the rate-limiting step in anaerobic digestion^5^.

Improvement of performance by altering process configurations have been successful^6,7^. However, the microbial consortium is the “core” of an anaerobic digester and broadly encompasses four metabolically diverse microbial communities involved in the degradation of complex organic substrates to methane and carbon dioxide; these include, the hydrolytic bacteria, acidogenic bacteria, acetogenic bacteria and the methanogenic archaea^2,8^. Each of these microbial communities share a cooperative relationship and are inter - dependent for growth and metabolic functions, this implies that interspecies microbial communication is indispensable among the anaerobic microbial communities involved in anaerobic digestion.

Conventionally, it has been accepted that within an AD system, bacterial “cross - talk” with the methanogenic archaea is generally via quorum sensing and the exchange of diffusive carriers such as hydrogen and formate^2,9,10^; in the presence of (semi)conductive nanoparticles^11^, however, a recently discovered phenomenon, Direct Interspecies Electron Transfer (DIET) has been shown to facilitate the direct exchange of electrons among bacteria as well as between bacteria and archaea via microbial nanowires in defined co - cultures and wastewater digester aggregates^11–15^. The structure and function of these microbial nanowires has been subject to contention^16–20^, however, recent investigations have clarified that these nanowires may be categorized as primarily extensions of the cytochrome-rich outer membrane and periplasm as well as type IV pili in *Geobacter sp*. which facilitate extra-cellular electron transport between bacteria and metal oxides^13^. Examples of microbial nanowire producing organisms include species of *Geobacter* such as *Geobacter sulfurreducens*, *Geobacter metallireducens* as well as *Shewenella oneidensis* ^14,21,22^.

Among diverse but interdependent microbial communities, intra – and – inter - species microbial communication has been thought to occur primarily through quorum sensing, production of membrane vesicles, pili and nanotube mediated communication^23–26^. Parasitism, mutualism or cooperative microbial interactions largely depend on the production and detection of signaling molecules by the interacting microorganisms^26,27^. In a hostile environment, the loss of signaling molecules will likely prove detrimental to these microbial communities. In such a scenario, the advantages of direct cell-cell contact confers a more positive fitness to the microorganisms which could result in a more robust and coordinated response to the environmental conditions. Among microorganisms, direct cell-to-cell interactions have been shown to occur via pili^13^ and more recently, interconnecting nanotubes^25^. Bacteria have also been shown to exchange nutrients and distribute metabolic functions among interconnected cells and auxotrophic mutants through these nanotubes^28^. Approaches based on the use of ad hoc co-cultures to investigate inter-microbial cytoplasmic exchange of cellular material through nanotubes have been demonstrated^25,28,29^, however, evidence of the same in environmental samples that has not been subject to genetic modification is lacking.

This study presents evidence of interspecies nanotube establishment among microorganisms in an anaerobic sludge inoculum digesting cellulose. This observation suggests that nanotube - mediated communication in the environment may be more prevalent than expected. Furthermore, it is also indicative of a microbial shift toward multi-cellularity under adverse extracellular conditions. This study has shown that in relatively unfavorable environments, microbial stress can induce the production of intercellular nanotubes among the anaerobic sludge microbiota. This discovery furthers understanding of microbial communication during the anaerobic digestion process with implications on process design, monitoring and operations. Additionally, the results of this study suggest that use of Iron Oxide Nanoparticles (IONp) could result in the significant improvement of hydrolysis and enhanced syntrophy among the microbial communities involved in anaerobic digestion.

## Results

### Evaluation of the Anaerobic Digestion Performance

**Figure 1:**
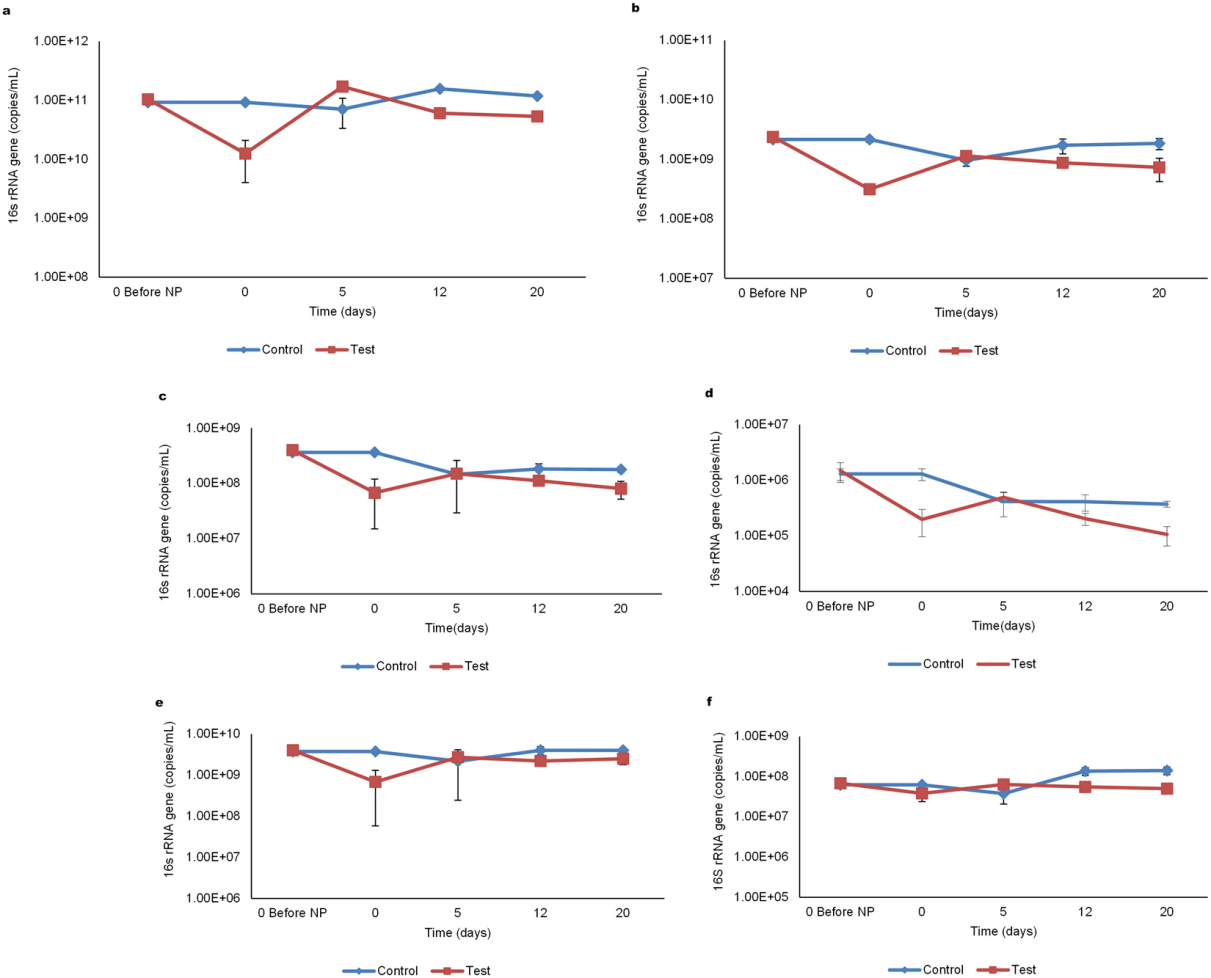
Microbial community shifts throughout anaerobic digestion. Overview of the shifts in the anaerobic sludge microbial communities throughout anaerobic digestion. Each of the data points represent the average values of four sample sets, comprised of two duplicate analysis from each of the duplicate reactor sets (Blank, Iron Blank, Control and Test). The error bars represent the standard deviation values among each of the reactor sets. (**a**,**b**) Depict changes in the 16S rRNA gene copy numbers of *Eubacteria* and *Methanogens* in the control and test reactors throughout anaerobic digestion. (**c**–**f**) Changes in the 16S rRNA gene copy numbers of *Methanosaetaceae*, *Methanosarcinaeceae*, *Methanobacteriales* and *Methanomicrobiales* in the control and test reactors throughout anaerobic digestion.

**Figure 2:**
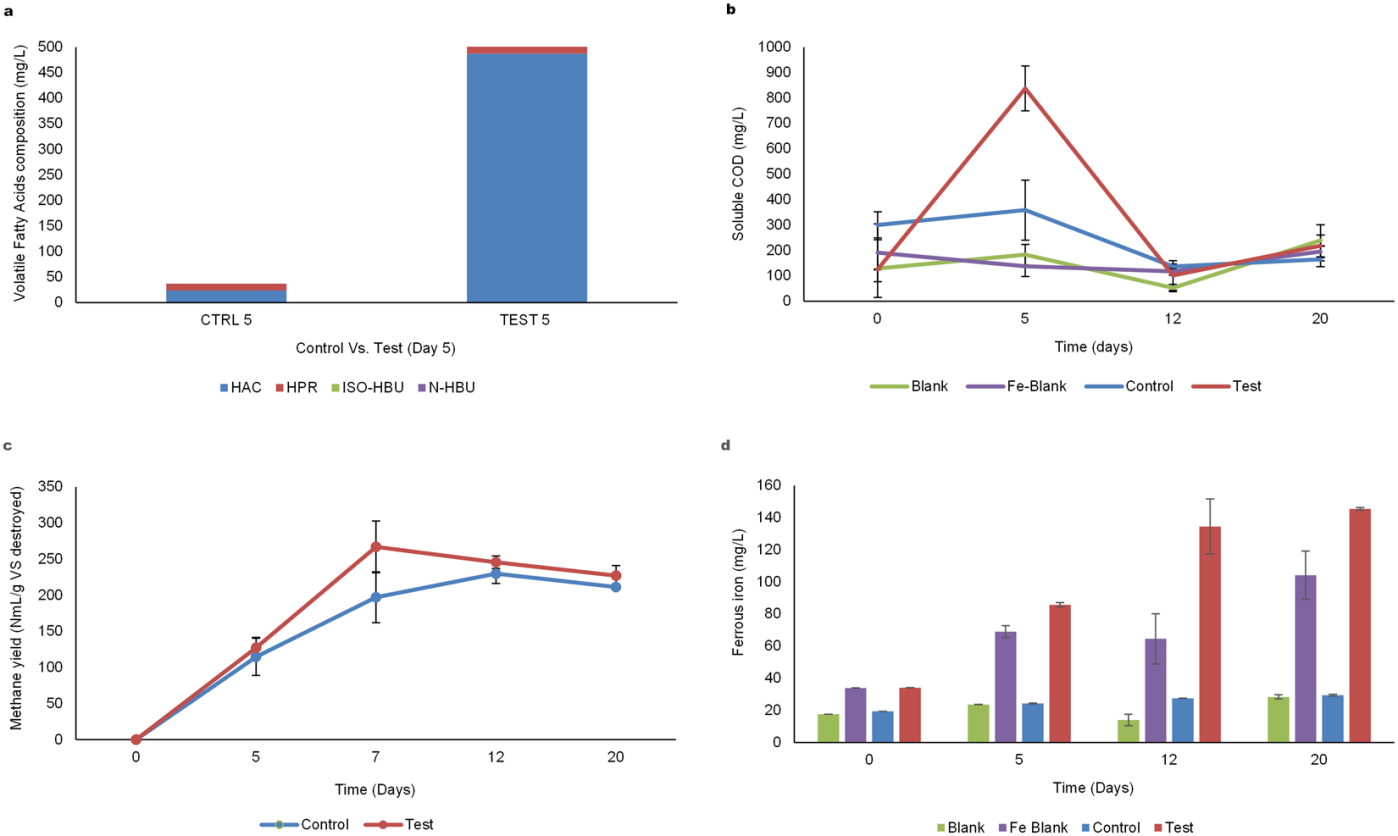
Anaerobic digestion process results. Overview of the anaerobic digestion process from day 0 till day 20. Average values of each reactor set (Blank, Iron Blank, Control and Test) is presented. The error bars represent the standard deviation between each duplicate reactor set. (**a**) Profile of the volatile fatty acids detected on day five of anaerobic digestion in the control and test reactors. (**b**) Overview of the changes in soluble COD detected in each of the representative reactor sets during anaerobic digestion. (**c**) Illustrates the difference in methane yield per gram volatile substance destroyed during anaerobic digestion. The volume of methane from each reactor was subtracted from the blank. The error bars were defined as the standard deviation between each duplicate reactor test set. (**d**) Overview of the changes in ferrous iron (Fe²^+^) concentration estimated during anaerobic digestion. The average values of Fe²^+^ estimated in each replicate reactor set during AD is presented. The error bars were defined as the standard deviation between each duplicate reactor set.

Supplementation of the anaerobic batch reactors digesting cellulose with iron oxide nanoparticles (IONp) resulted in a slight lag in methane production from the test reactors in comparison to the control. This event was likely due to the increased biomass stress exerted on the anaerobic sludge microbiota by the iron oxide nanoparticles which resulted in loss of microbial cell numbers within the test reactors (Fig. 1a,b). However, on day five, a drop in pH from 8.1 to 7.1, indicative of increased hydrolytic and acidogenic activity, was noted within the test reactors, whereas, within the control reactors the change in pH was not significant (Table 1). Increased fermentation and acidogenic activity has been shown to result in increased hydrogen production which in turn can disrupt the interspecies metabolic balances among the microbial communities present in the anaerobic sludge^18^. Therefore, the scavenging of hydrogen by the hydrogenotrophic methanogens via microbial syntrophy is important for the maintenance of the bio-process stability. Our results suggest that microbial syntrophy was enhanced in the presence of IONp, as shown by the negligible amounts of propionate (Fig. 2a) and hydrogen gas detected within the test reactors. The methane and carbon dioxide compositions of the biogas produced during anaerobic digestion has been presented in (Table 2).

**Table 1:** Changes in pH during anaerobic digestion.

Day	Blank	Fe Blank	Control	Test
0	7.8	8	7.9	8.1
5	8.0	8	7.5	7.1
12	7.4	7.7	6.8	7.0
20	7.4	7.6	6.9	7.0

**Table 2:** Methane and carbon dioxide composition of the biogas from each of the reactor sets during AD.

Day		BL 1	BL 2	FEBL 1	FEBL 2	CC 1	CC 2	CT 1	CT2
0	CO_2_	0	0	0	0	0	0	0	0
	CH_4_	0	0	0	0	0	0	0	0
5	CO_2_	5.2	1.8	2.9	3.7	39.9	28.6	50.4	47.1
	CH_4_	18.3	8.6	11.6	11.5	58.9	53.5	46.6	51.4
12	CO_2_	3.7	4.1	3.8	2.4	42.6	39.4	30.6	29.6
	CH_4_	13.3	20.5	18.9	14.5	61.9	64.5	74.3	71.5
21	CO_2_	6.3	6.6	6.8	4.7	33.4	23.6	22.4	11.2
	CH_4_	20.1	20.2	29.7	22.5	60	44	66.3	41

Enhanced microbial syntrophy within the test reactors was followed by an improvement in methane production, together with a significant increase (p < 0.05) in soluble COD from 124.9 mg/L on day zero to 837.5 mg/L (Fig. 2b) and a 20 fold increase in acetate concentration by day five. The acetate concentration was determined to be 487.25 mg/L within the test reactors and 23.65 mg/L in the control reactors (Fig. 2a). Analysis of the test reactor samples revealed a significant increase in *Eubacteria* (p < 0.0002) (Fig. 1a) followed by the *Methanogens* (p < 0.0003) (Fig. 1b). Among the *Methanogen* communities, the largest increase in 16S rRNA gene copy numbers was noted in the hydrogenotrophic order *Methanomicrobiales*, closely followed by *Methanosarcinaceae*, *Methanosaetaceae* together with a minor increase in *Methanobacteriales* (Fig. 1c–f). Between the two acetoclastic families, *Methanosarcinaceae* and *Methanosaetaceae*, the growth rate of *Methanosarcinaceae* has been shown to exceed *Methanosaetaceae* at higher acetate concentrations^30^, our experiments indicated similar results between the two acetoclastic families within the test reactors.

A drop in pH from 7.5 to 6.8 on day twelve within the control reactors signaled the establishment of acidogenesis accompanied by a net increase in 16S rRNA gene copy numbers across all the microbial communities. This suggested a slower acclimation process (more than twice the time required by the iron oxide nanoparticles-supplemented test reactors). In contrast, a decline in 16S rRNA gene copy numbers was noted within the test reactors, possibly due to substrate depletion (Fig. 1).

**Figure 3:**
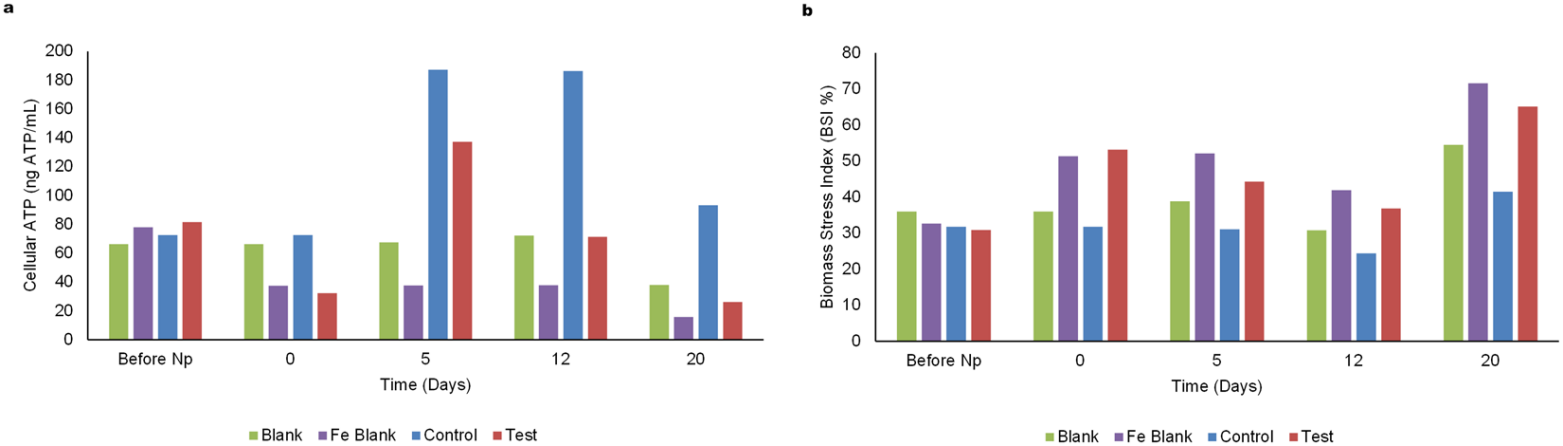
Adenosine Triphosphate (ATP) analysis. (**a**) Estimation of cellular ATP during anaerobic digestion. Overview of the changes in cellular ATP (ng ATP/mL) estimated in each of the reactors (Blank, Iron Blank, Control and Test) throughout anaerobic digestion. The cellular ATP represents the amount of ATP contained within the living cells and is a direct measure of the total living biomass within the reactor. The ATP results represent the mean values of each replicate reactor set. The error bars represent the standard deviation between each replicate reactor set. (**b**) Estimation of the Biomass Stress index. Overview of the changes in Biomass Stress Index (BSI%) estimated within each reactor set (Blank, Iron Blank, Control and Test). The BSI is indicative of the microbial stress level (quality) of the microbiota present. A BSI% above 50% indicates severe microbiological stress. The BSI% results represent the mean values of each replicate reactor set. The error bars represent the standard deviation between each replicate reactor set.

Taken together, our results show that within the control reactors, initial metabolic activity from day zero to day five was limited, suggestive of impaired hydrolysis and microbial adaptation, which in turn restricted carbon flow to the subsequent microbial communities involved in AD and resulting in the net decrease of 16S rRNA gene copy numbers. Whereas, in the test reactors, the addition of IONp did, after the initial lag, enhance cellulose hydrolysis which made available substrates for the acidogen, acetogen and methanogen communities (Figs. 1 and 3a) while simultaneously enhancing microbial syntrophy which resulted in an increase in 16S rRNA gene copy numbers across all the microbial communities as well as improved anaerobic digestion.

### Impact of Iron Oxide Nanoparticles (IONp) on the anaerobic sludge microbiota

Results from previous experiments had suggested that iron oxide nanoparticles could be cytotoxic to microorganisms. However, different studies have indicated contradictory results with regard to the use of iron oxide nanoparticles in wastewater treatment^11,31–39^. Therefore, in order to conclusively examine the effect of iron oxide nanoparticles on the anaerobic sludge microbiota, a combination of second generation ATP tests (to monitor the cellular ATP (cATP) and Biomass Stress Index (BSI%)) in conjunction with quantitative RT-PCR (to evaluate the effect of IONp on the individual microbial communities (*Methanogens*, *Eubacteria*, *Methanobacteriales*, *Methanomicrobiales*, *Methanosaetaceae* and *Methanosarcinaeceae*) within the anaerobic sludge microbiota) as well as a combination of Field Emission Scanning Electron Microscopy (FE-SEM) and Atomic Force Microscopy (AFM) was used to study the interaction between the iron oxide nanoparticles and anaerobic sludge microbiota.

**Figure 4:**
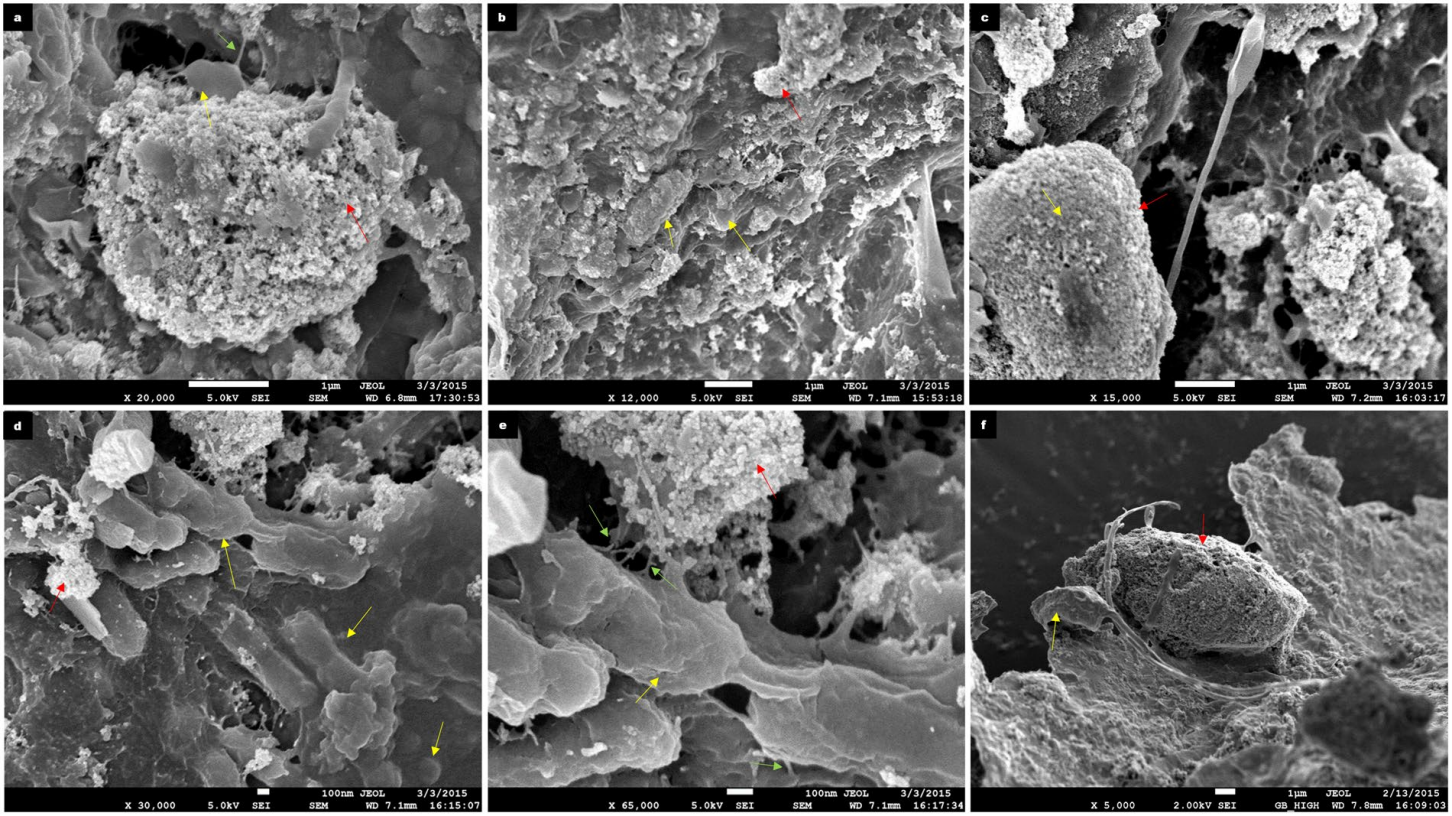
Cytotoxicity caused by iron oxide nanoparticles deposition. (**a**–**c**) FE-SEM images of day 20 samples extracted from the test reactor. The micrographs reveal extensive iron oxide nanoparticle deposition on the microbial cells which had resulted in mild to severe distortion of the cellular surfaces. Pointers indicate microbial cells (yellow), iron oxide nanoparticles (red) and intercellular appendages (green). Scale bar represents 1 µm. (**d**,**e**) FE-SEM images of day 20 samples extracted from the test reactor. Here, the microbial cells is seen to be extruding nanotubular structures (green), the microbial cell appears distorted, however the cellular integrity appears uncompromised. Furthermore, some microbial cells appear less susceptible to nanoparticle surface deposition while others have been heavily coated with the nanoparticles. Pointers indicate microbial cells (yellow), iron oxide nanoparticles (red) and intercellular appendages (green). Scale bar represents 100 nm. (**f**) FE-SEM images of day 20 samples extracted from the test reactor. In this image, a bacterial cell appears resistant to nanoparticle deposition in contrast to the adjacent cell which has been coated with nanoparticles. Pointers indicate microbial cells (yellow) and iron oxide nanoparticles (red). Scale bar represents 1 µm.

**Figure 5:**
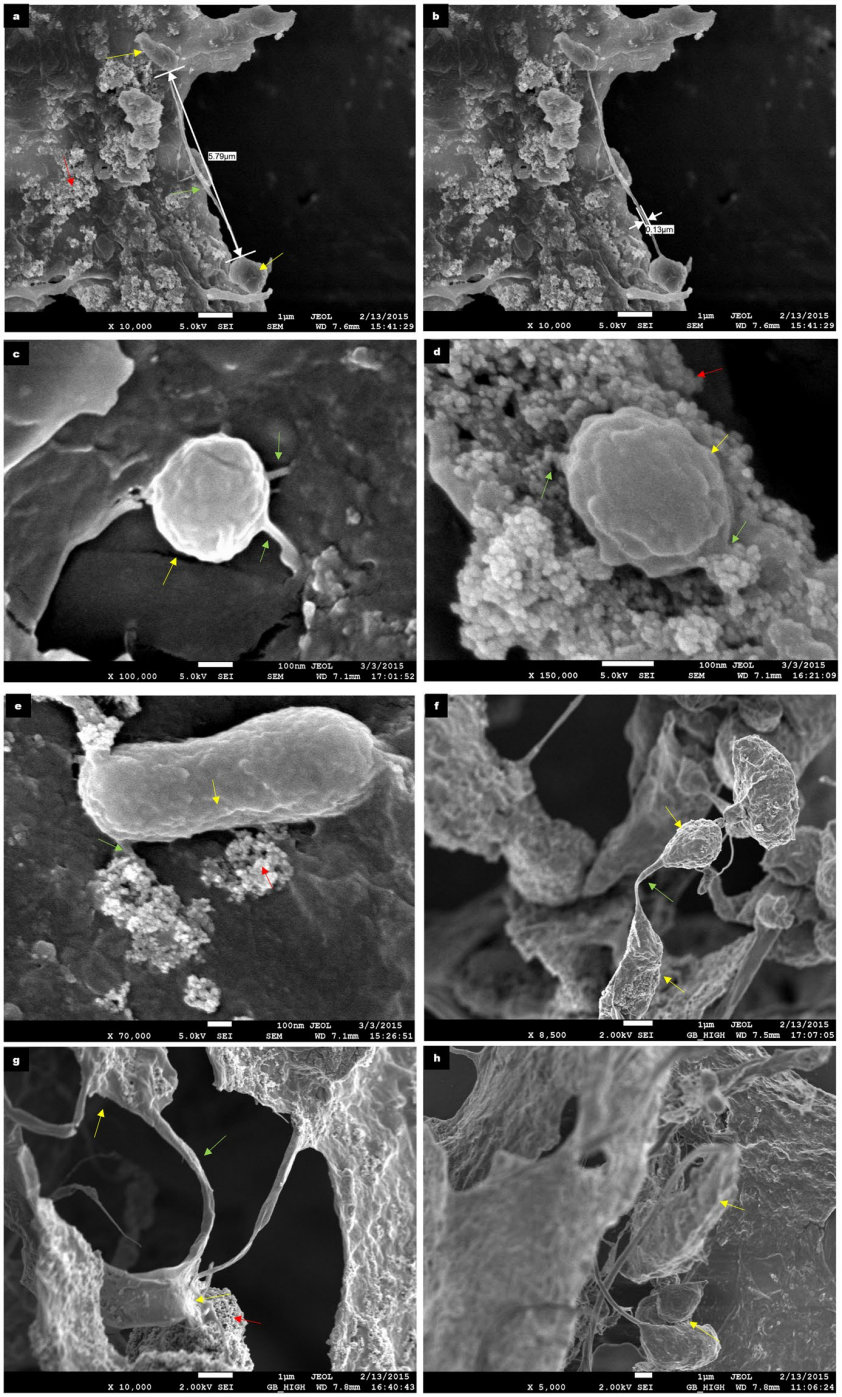
Formation of intercellular nanotubes between cells. Intercellular nanotubes visualized by FE-SEM in both the control and test reactors during anaerobic digestion. Pointers indicate microbial cells (yellow), iron oxide nanoparticles (red) and intercellular appendages (green). (**a**,**b**) Depicts a coccus and bacillus interconnected via interspecies nanotube in a sample taken from the test reactor on day 12. The length of the nanotube measured was approximately 5.79 µm and the diameter of the nanotube was found to be 0.13 µm. Scale bars represents 1 µm. (**c**) Depicts a coccoid microorganism in a day 5 sample extracted from a control reactor. (**d**) Depicts a coccoid microorganism embedded in iron oxide nanoparticles in a day 5 sample extracted from a test reactor. In both these images, the microbial cells appear healthy and no compromised by the iron oxide nanoparticles. Scale bars represents 100 nm. (**e**) Despite the toxicity of the IONp, this microbial cell appears healthy with surface deposition of nanoparticles not seen. Nanotubular structures can be seen emerging from different points of the microbial cell surface. The nanoparticles are present on cellular appendages. Scale bar represents 100 nm. (**f**) In this FE-SEM image of a sample taken from the test reactor on day 20, two morphologically distinct microbial cells appear to be interconnected by a nanotubular structure. Scale bar represents 1 µm. (**g**,**h**) FE-SEM images of samples taken from the test reactor on day 20. Extracellular microbial appendages are seen emerging from the cells. Scale bar represents 1 µm.

Our results indicate that augmentation of the anaerobic digestion system with iron oxide nanoparticles (IONp) had initially resulted in cellular stress due to the deposition of nanoparticles on the microbial cell surfaces. Scanning Electron micrographs of the sludge aggregates revealed slight to severely distorted microorganisms in the IONp supplemented test reactors (Fig. 4). The microbial cell surface deposition of the iron oxide nanoparticles likely resulted in cytotoxicity and cell death. However, it was also noted that deposition of iron oxide nanoparticles on the surface of the microbial cells was not uniform and a small proportion of the microbial biomass seemed “resistant” to IONp deposition (Figs 4d,f and 5d,e). It was not determined in this study if this was due to the differences in cell surface properties, such as differences in surface charges of the microbial cells.

Despite the significant decreases in 16S rRNA gene copy numbers across all the microbial communities following the addition of iron oxide nanoparticles; by day five of the anaerobic digestion process, the test reactors revealed a significant increase (p < 0.0002) in the *Eubacteria* 16S rRNA gene copy numbers (1.25 × 10¹° to 1.71 × 10¹¹) as well as Methanogen (p < 0.0003) 16S rRNA gene copy numbers (3.18 × 10^8^ to 1.14 × 10^9^). Within the methanogenic community, the hydrogenotrophic *Methanomicrobiales* recorded the largest increase in gene copy numbers from 6.80 × 10^8^ to 2.74 × 10^9^ (p < 0.001) followed by the acetoclastic *Methanosarcinaceae* (Fig. 1f,d). In contrast, quantitative PCR analysis of samples from the control reactors had shown a net decrease in 16S rRNA gene copy numbers across all the microbial communities. Consistent with this data, cellular ATP evaluations also revealed a 324.8% increase within the test reactors (Fig. 3a). Interestingly, Fe²^+^ iron in the test reactors had simultaneously increased by 128% indicative of the presence of a dissimilatory iron reducing microbial community; in comparison Fe²^+^ iron increase in the control reactors was found to be negligible (Fig. 2d).

**Figure 6:**
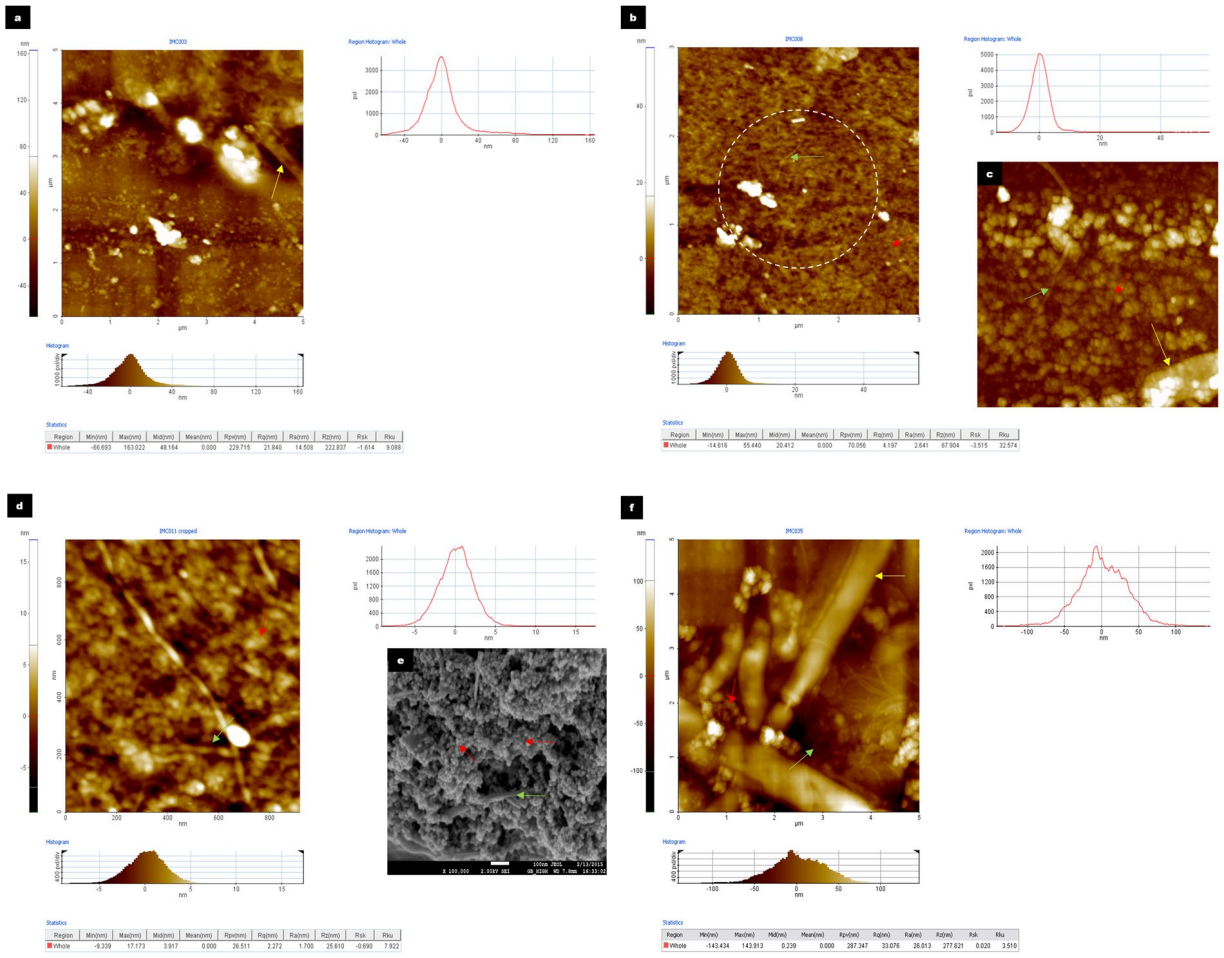
Comparison of Atomic Force Microscopic images of samples taken from the Control and Test reactors. Pointers indicate microbial cells (yellow), iron oxide nanoparticles (red) and intercellular appendages (green). (**a**) Represents the day 8 sample analyzed from the control reactor. Here mainly microcrystalline cellulose and microbial cells are seen. Scale bar represents 3 µm. (**b**) Represents the day 8 sample analyzed from the test reactor. Here, primarily IONp are seen with microbial wire-like appendages interspersed among the nanoparticles. Scale bar represents 3 µm. (**c**) Represents a magnified AFM image depicting the morphology of the iron oxide nanoparticles and cellular structures. Scale bar is 850 nm. (**d**) Represents a magnified AFM image depicting the morphology of the iron oxide nanoparticles and cellular structures. Scale bar is 850 nm. (**e**) FE-SEM image of iron oxide nanoparticles with wire-like cellular appendages, detected in a sample extracted from the test reactor on day 8 of anaerobic digestion. Scale bar represents 100 nm. (**f**) Represents Atomic Force Microscopic images of a day 5 test sample showing healthy microbial cells. Scale bar represents 5 µm.

Scanning electron micrographs and Atomic Force microscopy revealed the presence of two structurally distinct microbial appendages resembling microbial nanowires^13^ (Fig. 6b) as well as interspecies nanotubes in samples taken from the test reactors^25,28^ (Fig. 5). In comparison, only microbial structures resembling intercellular nanotubes were detected in samples taken from the control reactors. Previous studies have shown that Dissimilatory Iron Reducing Bacteria (DIRB) produce pili-like appendages which can function as microbial nanowires in the presence of solid iron oxide aiding in iron reduction^12,13^. Furthermore, it was observed that the structural integrity of the microbial nanowire - producing cells appeared largely uncompromised by the presence of iron oxide nanoparticles. These microbial appendages were predominantly embedded among the iron oxide nanoparticles (Fig. 6c,d). This observation suggested that these cellular structures may have been involved in dissimilatory iron reduction within the test reactors. Additionally, the supply of Fe²^+^ within the test reactors, which has been shown to be important for microbial biosynthesis pathways and enzyme repair functions by the DIRB activity (Fig. 2d) could have contributed to the overall enhanced AD performance despite the initial loss of microbial cells due to nanoparticle cytotoxicity.

### Microbial stress and intercellular nanotubes

**Figure 7:**
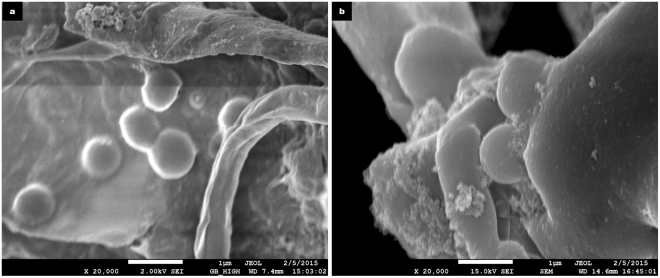
Anaerobic sludge inoculum. (**a**,**b**) FE-SEM images of samples extracted from the Blank reactor and Iron-Blank rectors respectively on day 0 of anaerobic digestion. In both the micrographs, no nanotubes are visible, indicative that the nanotubes were produced in response to biomass stress in the individual reactors as the anaerobic digestion progressed. Scale bars represent 1 µm.

FE-SEM analysis of the anaerobic sludge inoculum on day zero did not reveal the presence of intercellular nanotubes (Fig. 7), this observation suggested that these microbial structures were not present at the start of anaerobic digestion. Continuous monitoring of methane production during the initial phase of anaerobic digestion revealed a slow start both in the control as well as test reactors. While the initial inhibition in methane production was expected due to IONp cytotoxicity within the test reactors, biomass stress had also been observed with the control reactors, this observation implied that the use of insoluble microcrystalline cellulose as the substrate for anaerobic digestion within both the control as well as test reactors could have been also one of the contributing factors to biomass stress.

Analysis of the biomass stress on day zero indicated relatively high BSI values at 31.8% in the control and 30.9% in the test reactors which further increased by 72.2% shortly after IONp addition to a final value of 53.2% on day zero in the test reactors. Biomass stress measured within the Iron blank reactors (containing only anaerobic sludge inoculum and iron oxide nanoparticles) similarly recorded an increase in BSI from 32.7% (before nanoparticle addition) to 51.4% (after nanoparticle addition) on day zero, this clearly demonstrated the initial cytotoxicity of the iron oxide nanoparticles on the anaerobic sludge microbiota (Fig. 3b).

By day five, however, while BSI in the control reactors had remained largely unchanged at 31.1%, the BSI had decreased by 16.7% in the test reactors to 44.3% indicative of microbial adaptation and cell recovery (Fig. 3b). These results were further corroborated by the estimation of cellular ATP, where the cATP in the test reactors had initially decreased from 81.6 ng ATP/mL (before IONp addition) to 32.3 ng ATP/mL (after IONp addition) on day zero. Thereafter, cATP had increased by 324.8% to 137.2 ng ATP/mL by day five. Furthermore, quantitative RT-PCR data also indicated significant increases in the 16S rRNA gene copy numbers in both the *Eubacteria* (1268%) as well as the *Methanogen* communities (258.5%). In our study, this event has been referred to as the microbial rewiring and cell recovery phase. The anaerobic process performance had also improved in tandem.

**Figure 8:**
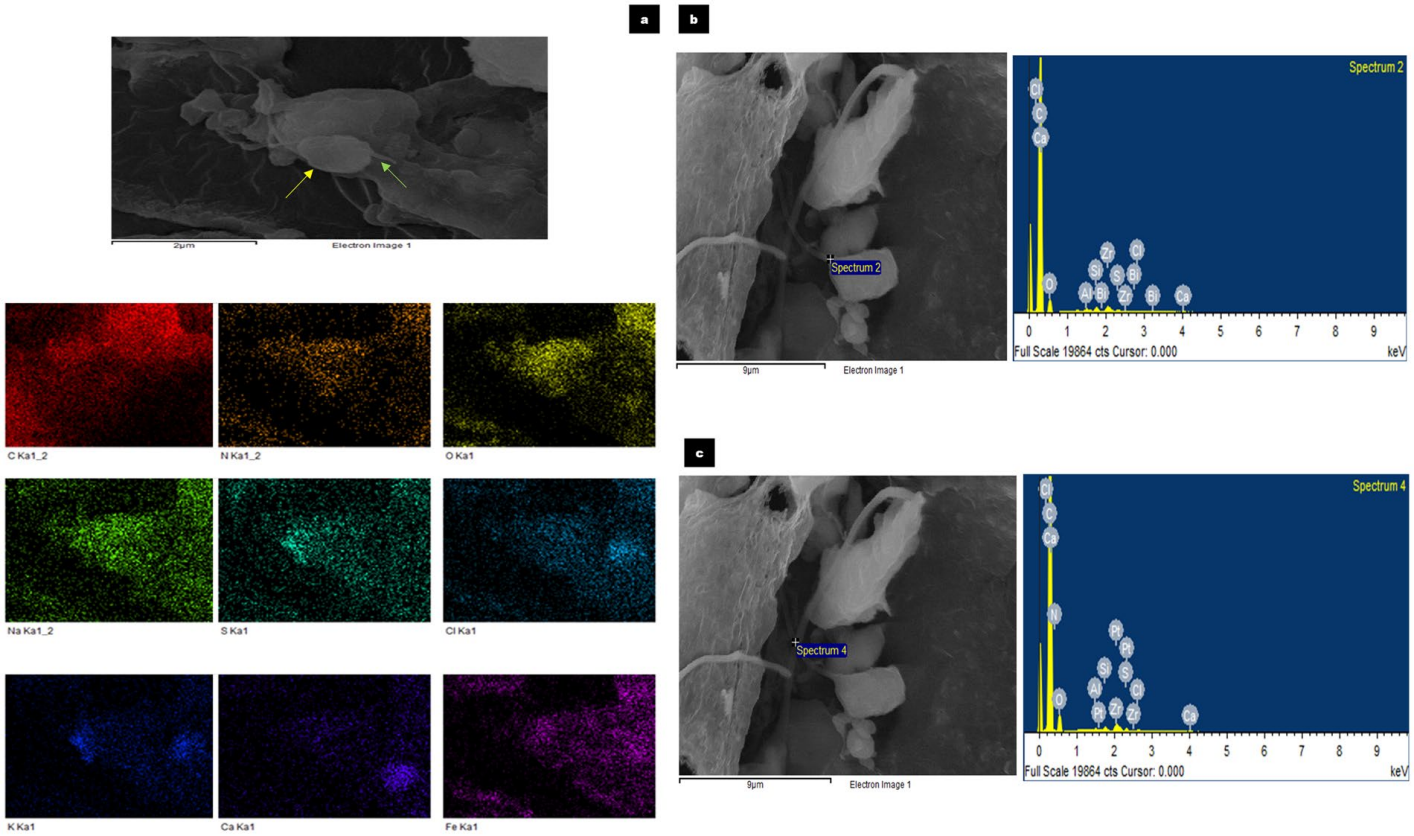
Elemental mapping of the microbial cells and appendages. The following images represent the elemental mapping that was performed using an Energy Dispersive X-ray spectrometer (EDX) (Oxford Instrument, X-max, 80 mm^2^) attached to the FE-SEM to determine elemental compositions of the microbial cells as well as extracellular appendages. Pointers indicate microbial cells (yellow), iron oxide nanoparticles (red) and intercellular appendages (green). (**a**) Elemental mapping of the image and the representative elements (C, N, O, Na, S, Cl, K, Ca and Fe) detected in day 12 test reactor sample. Scale bar represents 2 µm. (**b**,**c**) In this day 12 control reactor sample, the individual points at which EDX spectra were obtained has been highlighted and the respective elemental compositions are shown in the adjacent graphs. The results indicate that the appendages are cell derived and have similar elemental compositions. Scale bar represents 9 µm.

Nanotube – mediated bacterial communication has been thought to represent a significant form of direct cell - cell communication in microbial communities although evidence to support this has been lacking in anaerobic digestion systems. In this study, improved performance despite the relatively high BSI, had led to the hypothesis that intercellular nanotube mediated communication may have played a significant role in the anaerobic digestion process. Supporting this hypothesis, scanning electron micrographs had revealed the presence of interspecies nanotubes interconnecting the microorganisms within both the control as well as test reactors from day 5 till the end of anaerobic digestion on day 20. The concentration of nanotubular structures was found to be higher in the test reactors in comparison to the control. The diameters of the nanotubes were determined to be between 0.13 µm and 0.47 µm. Elemental mapping through Energy Dispersive X-ray Spectrometer (EDX) revealed that the elemental compositions of the bacterial cells and the interconnecting nanotubes were similar (Fig. 8a–c). This indicated that the tubular structures were cell - derived as previously reported^25^. Given the relatively high BSI in the control as well as test reactors, this study would suggest that an environment leading to microbial stress was a key inducer for the production of intercellular nanotubes.

It has been reported that microbial phenomena such as development of bacterial competence^40^, quorum sensing^41^, bioluminescence^42^, and rapid increase in cell density is preceded by an initial lag phase, this phenomenon was also observed in this study - where an initial lag phase preceded a rapid increase in microbial numbers. This study would propose that this lag phase could also be a characteristic feature of intercellular nanotube formation. In conclusion, this study has shown that interspecies nanotubes among microorganisms can also be formed among the anaerobic sludge microbiota subjected to microbial stress as well as shear forces with intermittent bioreactor stirring of 50 rpm.

## Discussion

We investigated the impact of iron oxide nanoparticles on anaerobic sludge microbiota during the anaerobic digestion of cellulose. Cytotoxic stress and loss of microbial numbers as a result of iron oxide nanoparticle addition was reversed as anaerobic digestion progressed in the iron oxide nanoparticles-augmented test reactors. Establishment of a dissimilatory iron reducing bacterial community may have aided in the recovery of *Eubacteria* and *Methanogens* through the supply of Fe²^+^ which had resulted in improved enzymatic activitiy^35^. Additionally, it is thought that the IONp had increased the surface area of contact between the hydrolytic enzyme complex and the substrate which resulted in enhanced hydrolysis of the insoluble microcrystalline cellulose^43^. This increased the bioavailability of substrates to the acidogen, acetogen and methanogen communities and enabled microbial cell recovery. Microbial syntrophy was also enhanced in the presence of IONp which created a conducive environment for the anaerobic digestion process. Therefore, this study would conclude that the addition of iron oxide nanoparticles to the anaerobic digestion of cellulose had an overall ameliorative effect despite the initial cytotoxicity of the nanoparticles. Our results indicate that hydrolysis /and acidogenesis was improved and microbial syntrophy enhanced in the presence of IONp. Furthermore, the results of this study also establish three distinct microbial phases following addition of the nanoparticles: microbial stress and cell death of approximately one log order of magnitude, followed by microbial rewiring, and recovery.

Nanotube - mediated bacterial communication was first demonstrated by Dubey and Ben-Yehuda in 2011, it was shown to facilitate exchange of cytoplasmic contents between identical as well as different species of bacteria^25^. Subsequent studies have reported that metabolic cross-feeding^28^ is facilitated between bacteria interconnected by intercellular nanotubes, and nutritional stress has also been reported to induce nanotube formation to facilitate cytoplasmic exchange and energetic coupling between the interconnected microorganisms^29^. Under adverse environmental conditions, direct cell-cell contact ensures the targeted exchange of metabolic products between microbial cells, mitigating the loss of metabolic products to the environment as well as ensuring synergistic growth and activity^28^. In this study, unfavorable environmental conditions had led to biomass stress which resulted in the establishment of intercellular nanotubes within the anaerobic sludge microbial community. Evaluation of this study’s results would suggest that microorganisms had preferentially established communication through intercellular nanotubes under influence of unfavorable environmental conditions. Given the improved performance of the test reactors despite the initial cytoxicity of the iron oxide nanoparticles, it was likely that intercellular nanotube – mediated communication had played an important role in anaerobic digestion. This study has shown evidence of the spontaneous establishment of intercellular nanotubes within the anaerobic sludge microbiota, thus advancing previous knowledge of intercellular nanotubes formed in engineered and ad hoc microbial cultures. Future studies should focus on determining the importance and prevalence of nanotube-mediated microbial communication in different environmental systems. The microbial transition from uni-cellularity to multi-cellularity via interspecies nanotubes under unfavorable conditions could have significant implications both in anaerobic digestion as well other microbial processes.

## Materials and Methods

### Inoculum and Substrate

The anaerobic sludge inoculum used in this experiment was collected from a mesophilic anaerobic digester treating primary and secondary municipal sludges. The sludge characteristics were as follows: Total Solids = 12.53 g/L, Volatile Solids = 9.31 g/L, Total Suspended Solids = 12.2 g/L, Volatile Suspended Solids = 9.1 g/L and pH = 7.5. The anaerobic seed was stored in a cold room for no more than 5 days and degassed^44^ prior to inoculation into the anaerobic digestion batch reactors.

The substrate was insoluble microcrystalline cellulose (Sigma Aldrich), 3.4 g cellulose/L water was added to each of the reactors. In general, cellulose is considered the substrate of choice in the study of hydrolysis^45,46^; since the complete anaerobic degradation of cellulose closely simulates the conventional AD bioprocess^47^ including hydrolysis, acidogenesis, acetogenesis and methanogenesis. The individual reactor compositions have been listed in Table 3.

**Table 3:** Reactor compositions.

Content/Reactor	Blank (BL)		Fe-Blank (Fe-BL)		Control (CC)		Test (CT)	
	BL1	BL2	Fe-BL1	Fe– BL2	CC 1	CC 2	CT 1	CT2
Substrate –Microcrystalline Cellulose (Grams)	0	0	0	0	1.53	1.53	1.53	1.53
Inoculum (mL)	140	140	140	140	140	140	140	140
Deoxygenated water (mL)	310	310	210	210	310	310	210	210
Iron oxide nanoparticle suspension (mL)	0	0	100	100	0	0	100	100
Total volume (mL)	450	450	450	450	450	450	450	450

### Process operation

The effect of iron oxide nanoparticles on the anaerobic digestion of microcrystalline cellulose was evaluated through the BMP test using the Automatic Methane Potential Test System (AMPTS II) (Bioprocess Control AB, Sweden), designed for the automatic, real–time monitoring of methane production during anaerobic digestion. Batch AD of cellulose was carried out at 35 °C ± 1 °C under standard pressure for 20 days after which methane production stopped. The standard protocols for the AMPTS II (Bioprocess Control AB, Sweden) were used in this study. Two reactors were used for each set of tests. These were: (i) Control (C1-C2) set which contained 140 mL of anaerobic inoculum sludge, 1.53 grams microcrystalline cellulose powder mixed in 210 mL of deoxygenated water and 100 mL of deoxygenated water to obtain a final working volume of 450 mL, (ii) Test reactors (T1-T2) which contained 140 mL anaerobic inoculum sludge, 1.53 grams microcrystalline cellulose powder mixed in 210 mL of deoxygenated water and 100 mL of synthesized iron oxide nanoparticle suspension and, (iii) Blank reactors (BL1-BL2) which comprised 140 mL anaerobic sludge and 310 mL deoxygenated and Iron oxide nanoparticle Blank reactors (FeBL1-FeBL2) which contained 140 mL anaerobic sludge, 210 mL deoxygenated water and 100 mL of synthesized iron oxide nanoparticle suspension. The total volume of each AMPTS batch reactor was 600 mL and the reaction volume was 450 mL. Each of the reactors were connected to the carbon dioxide absorption unit and flushed with nitrogen gas for 2 minutes each to maintain an anaerobic environment. The batch reactors were subsequently connected to the on-line gas monitoring system. Details of the reactor system is as shown in Table 3. No pH amendment or nutrient solutions were added to mitigate possible interactions with the iron oxide suspension. The stirring speed was 50 rpm. Each sample set was tested in duplicate. The average values of results from each of the reactor sets are reported in this study.

### Analytical methods

Characterization of the sludge samples was conducted in accordance with the standard methods^48^. VFA analysis of acetic, propionic, n-butyric, iso-butyric, n-valeric, iso-valeric and n-caproic acids was with an Agilent gas chromatograph system (6890 N) equipped with a flame ionization detector (FID) following Zhou *et al*.^49^. The various VFAs were separated with a 30 m × 0.32 mm × 0.50 µm DB - FFAP fused silica capillary column. The injector and detector temperatures were set at 260 °C and 300 °C respectively. The oven temperature was initially 60 °C, ramped up to 120 °C at a rate of 20 °C/min and held for 1 min. Finally, the temperature achieved 240 °C at 20 °C/min and held for 3 min. Helium was the carrier gas with a constant pressure of 103.0 kPa. The injector was running in a split mode with split ratio of 50:1. Prior to VFA analysis, test samples were withdrawn from the mixed liquor, centrifuged at 10,000 rpm for 10 minutes followed by filtration of the supernatant through a 0.2 micron syringe filter. The samples were then suitably diluted and fixed by the addition of 100 µL of 10% formic acid to 900 µL of the filtrate.

Composition of the biogas generated was determined using a Shimadzu GC-TCD fitted with a Porapak N column (1500 × 6.35 mm). The carrier gas was Helium set at a flow rate of 50 mL/min. The column, detector and injector temperatures were set at 28, 38 and 128 °C, respectively. The peak areas were calculated with a Shimazdu Chromatopac C-R6A integrator. 5 mL samples were withdrawn from the gas sampling ports in the reactors using a gas - tight Agilent glass syringe and the sample was injected into the instrument for analysis.

### Extraction and Quantification of Fe²^+^ iron and Total Fe³^+^ iron

Iron (Fe³^+^ and Fe²^+^) was extracted from sludge samples with the Hydrochloric acid extraction method^48^. Briefly, 2 mL of test sludge was mixed with 8 mL of 0.5 N hydrochloric acid taken in a capped test tube, 2 glass beads were added into the mixture to enhance mixing. The test tube was capped and incubated at room temperature on an orbital shaker for 30 minutes to facilitate the extraction of Fe³^+^ and Fe²^+^ iron from the sludge sample. The samples were then centrifuged at 10000 rpm for 10 minutes and the supernatant was filtered through a 0.2 μm syringe filter. The filtrate was used for quantification of Fe³^+^ and Fe²^+^ iron. The concentration of Fe³^+^ and Fe²^+^ iron was determined by the Phenanthroline Assay^50^.

### Preparation and Characterization of Iron oxide nanoparticles

A homogenous suspension of magnetite (Fe_3_O_4_) nanoparticles was synthesized by the co-precipitation method. An acidic solution containing 0.8 M FeCl_3_ and 0.4 M FeCl_2_ dissolved in 0.4 M HCl was added drop - wise with constant stirring into 1.5 M NaOH solution. A black precipitate of Fe_3_O_4_ was generated which was isolated in a magnetic field. The precipitate was washed with deoxygenated water, centrifuged, neutralized and finally suspended in deoxygenated water^51^. Nanoparticle characterization (size and morphology) was carried out on freeze - dried samples by Field Emission Scanning Electron Microscopy (FESEM) JEOL JSM - 7600F. The nanoparticles were uniform in shape distribution and almost spherical with an average diameter of 8.7 ± 2 nm. The nanoparticle structure was determined with the Bruker D8 Advance X-ray Diffractometer (XRD) with monochromated high intensity Cu Kα radiation (λ = 1.5418 Å).

### DNA sample preparation and extraction

Sludge samples were initially diluted with DI water. The diluted sludge samples were centrifuged at 10,000 rpm for 5 minutes and the pellet washed with phosphate buffered saline (PBS), this washing cycle was repeated three times to remove any impurities and inhibitory substances in the sample before DNA extraction. The washed pellet was then re-suspended in PBS and the final volume made up to 1 mL. 100 µL of this processed sludge sample was then loaded into an automated nucleic acids extractor (MagNa Pure Nucleic Acids Extractor, Roche, Germany). A standard pre - loaded nucleic acid extraction protocol was initiated. The final DNA elution volume was set at 100 µL. The extracted DNA was stored at −20 °C before further analyses.

### Real-time Quantitative Polymerase Chain Reaction

The abundance of the 16S rRNA gene of each of the microbial communities was quantified by RT-PCR on a Roche LightCycler 480 II system (Germany). DNA samples from each reactor was analyzed in duplicate and the average of four replicates from each reactor test set are reported in this study. Shifts in 16S rRNA gene copy numbers of the *Eubacteria*, two hydrogenotrophic orders – *Methanobacteriales* and *Methanomicrobiales* as well as an acetoclastic order comprising two families – *Methanosarcinaeceae* and *Methanosaetaceae* during AD was monitored. The primer/probe sequences of the target microbial communities were adapted from Yu *et al*.^52^ and are listed in (Supplementary Table 1). The probes as well as the forward/reverse primers were synthesized by TIB MolBiol (Berlin, Germany). Each well of the qPCR Lightcycler microplate contained a reaction mixture of 2 µL DNA template, 1 µL forward primer, 1 µL reverse primer, 2 µL TaqMan probe (community – specific), PCR – grade water as well as 10 µL of LightCycler mastermix (Roche, Germany). The final reaction volume in each well was 20 µL. All samples were analyzed in duplicates. The microplates were sealed and loaded into a Roche Lightcycler 480 – II (Roche Diagnostics, Mannheim, Germany) for the qPCR reaction. The construction of the standard curves are as described by Bialek *et al*.^53^.

A two-step amplification of the target DNA, combining the annealing and the extension steps, as described in a previous study^30^ with slight modifications was followed. Briefly, the 2 step protocol included an initial incubation step for the activation of Taq DNA polymerase at 95 °C for 10 minutes followed by thermal denaturation at 95 °C for 10 minutes (55 cycles) and simultaneous primer annealing and extension at 60 °C for 30 seconds. The emitted fluorescence signal data was recorded in the “single mode” and the results were processed with the Roche LightCycler Software version 4.0.

### Adenosine triphosphate (ATP) analysis

ATP is a direct, interference-free, microbiologically non-specific indicator of the total living biomass. QuenchGone21^TM^ Wastewater kits (LuminUltra Technologies Ltd. New Brunswick, Canada) which is based on the principle of second generation ATP technology was used to monitor total and dissolved ATP. The ATP was measured using the luciferase assay. A study sample containing ATP was introduced into a solution containing the Luciferase enzyme to produce a quantifiable light reaction. The light produced was detected in a luminometer as Relative Light Units (RLU) according to equation (1):

1 $$ATP+{O}_{2}+luciferin\mathop{\longrightarrow }\limits^{M{g}^{++}\,luciferase}AMP+PPi+oxyluciferin+light$$

ATP extraction and analyses was carried out according to standard kit instructions. Briefly, the assay steps included an ATP standard calibration assay (ATP1) that converted luminometer RLU values into real ATP concentrations. One calibration was carried out per test set. Using clean pipette tips, two drops of (100 µL) of Ultracheck 1(standardized ATP solution) was added to 300 µL of Luminase taken in a 12 × 55 mm assay tube. The mixture was gently mixed. 100 µL of the sample was loaded onto a microplate well and immediately inserted into a TECAN infinite 200 PRO microplate reader. The luminescence was measured. Each of the measurements were carried out in duplicates. The measured value was designated as RLU_ATP1_. *Total ATP (tATP) Analysis*: Total ATP (tATP) was defined as the measurement of all the ATP molecules within a sample, including ATP from living cells in addition to ATP that had been released from dead cells. Total ATP analysis of individual samples withdrawn from each of the triplicate reactors at different sampling time points was performed as follows, *Extraction*: The test sample was mixed well. Using a wide-mouth pipette tip, 1 mL of the sludge sample was added to 2 mL UltraLyse 30 (Extraction Tube). The tube was capped and inverted three times to ensure through mixing. This tube was then incubated for one minute to facilitate extraction. *Dilution*: The contents of the UltraLyse 30 (Extraction Tube) post incubation was poured into a new 8 mL UltraLute/Resin (Dilution) tube. The mixture was transferred back and forth between the two tubes several times to ensure through mixing. The Ultra Lute/ Resin (Dilution) tube beads were then allowed to settle. *Assay*: Using a clean pipette tip, 100 µL of the Ultra Lute/ Resin (Dilution) tube content was added to 300 µL of Luminase taken in a 12 × 55 mm test tube. The mixture was gently swirled, 100 µL was sample was withdrawn and loaded onto the prepared microplate, which was then inserted into the TECAN infinite 200 PRO microplate reader and luminescence was measured. Total ATP was computed according to equation (2):

2 $$tATP(\frac{ngATP}{mL})=\frac{RLUtATP}{RLUATP1}\times 11(\frac{ngATP}{mL})$$

#### *Dissolved ATP (dATP)analysis*

Dissolved ATP (dATP) was defined as a measurement of only the dead biomass. It was representative of the ATP present within a sample that had been released from dead cells. Dissolved ATP analysis of individual samples withdrawn from each of the triplicate reactors at different sampling time points was performed as follows, *Dilution*: Using a clean pipette tip, 100 µL of well-mixed anaerobic sludge reactor sample was added to 10 mL LumiSolve (Stabilizer) Tube. The tube was capped and inverted three times to allow through mixing. The sample was then incubated for one minute. *Assay*: Using a clean pipette tip, 100 mL of the LumiSolve (Stabilizer) Tube mixture containing the test sample was added to 300 µL of Luminase taken in a clean 12 × 55 mm assay test tube. The mixture in the test tube was gently swirled, 100 µL of the sample was extracted and loaded into a prepared microplate which was promptly inserted into the TECAN infinite 200 PRO microplate reader and luminescence was measured. Dissolved ATP was computed according to equation (3):

3 $$dATP(\frac{ngATP}{mL})=\frac{RLUdATP}{RLUATP1}\times 101(\frac{ngATP}{mL})$$

*Cellular ATP (cATP)* was defined as the ATP contained within the living cells and was a direct indicator of the living microbial population (quantity). It was defined by equation (4):

4 $$cATP(\frac{ngATP}{mL})=tATP(\frac{ngATP}{mL})-dATP(\frac{ngATP}{mL})$$

*Biomass Stress Index (BSI)* was indicative of the stress level experienced by the microbial population present within the test system and was used as a parameter to monitor the toxicity within the bioreactor. BSI was defined by equation (5):

5 $$BSI \% =\frac{dATP(\frac{ngATP}{mL})}{tATP(\frac{ngATP}{mL})}\times 100 \% $$

### Field Emission Scanning Electron Microscopy (FE-SEM)

Samples extracted from the bioreactors were immediately frozen at −80 °C for 30 minutes followed by freeze-drying under vacuum (Labconco 73820 FreezeZone Plus^TM^ 4.5 Liter Cascade Freeze Dry System, Kansas City, MO) at 0.021 mBar and −70 °C for 18 hrs −20 hrs.

The samples were mounted on a carbon tape and sputter-coated with Platinum (JEOL JCF-1600) prior to examination by Field-Emission Scanning Electron Microscopy (FE-SEM, JEOL JSM – 7600F) at 5 kV acceleration voltage. Elemental mapping was performed using an Energy Dispersive X - ray spectrometer (EDX) (Oxford Instrument, X-max, 80 mm^2^) attached to the FE-SEM.

### Cell imaging by Atomic Force Microscopy (AFM)

At selected data points, samples were extracted from the reactors and 0.5 mL of the sample was placed on a clean glass slide and gently smeared. The smear was air-dried and immediately observed using an Atomic Force Microscope (XE-100 AFM, Park Systems Corp., Suwon, Korea) Images were collected in ambient air in both contact and non-contact mode.

### Statistical Analysis

Two tailed Student *t*-test with equal variance was used to determine statistical significance in the bioprocess results between the control and test systems, as well as differences in the 16S rRNA gene copy numbers in the microbial populations between the control and iron oxide nanoparticles- augmented test reactors. α was defined as 0.05 in the formation of the null hypothesis. The confidence intervals on the difference between the means was adjusted to 95%. Results were considered statistically significant when p ≤ 0.05.  

## Electronic supplementary material

Supplementary Dataset 1
