# Supplementary material for: Microbial stress mediated intercellular nanotubes in an anaerobic microbial consortium digesting cellulose
Source: Sci Rep. 2017 Dec 21;7:18006. doi: 10.1038/s41598-017-18198-w (PMC5740137; doi:10.1038/s41598-017-18198-w)
Supplement: Supplementary file 1 — Supplementary Dataset 1 [file 41598_2017_18198_MOESM1_ESM.doc]

**Microbial stress mediated intercellular nanotubes in an anaerobic microbial consortium digesting cellulose**

Martina John^1^*, Antoine Prandota Trzcinski^1^, Yan Zhou^1^, Wun Jern Ng^1, 2^ *

^1^ Advanced Environmental Biotechnology Centre, Nanyang Environment and Water Research Institute, Nanyang Technological University, 1 Cleantech Loop, CleanTech One, #06-08, Singapore – 637141.

^2^ Division of Environmental and Water Resources, School of Civil and Environmental Engineering, Nanyang Technological University, 50 Nanyang Avenue, Singapore – 639798

Corresponding Authors: Wun Jern Ng ([WJNG@ntu.edu.sg](mailto:WJNG@ntu.edu.sg))

Martina John (martina1@e.ntu.edu.sg)

**List of Supplementary Figures**

**Table 1.** Composition of Taq man probe and primer sets

| **Set name** | **Target group** | **Oligonucleotide sequence (5’ → 3’)^b^** | **Amplicon size (bp)** |
| --- | --- | --- | --- |
| ARC787F  ARC915F  ARC1059R  BAC338F  BAC516F  BAC805R | *Archaea*    *Eubacteria* | F: ATTAG ATACC CSBGT AGTCC  T: AGGAA TTGGC GGGGG AGCAC  R: GCCAT GCACC WCCTC T  F: ACTCC TACGG GAGGC AG  T:TGCCA GCAGC CGCGG TAATA C  R:GACTA CCAGG GTATC TAATC C | 273  468 |
| MBT857F  MBT929F  MBT1196R | *Methanobacteriales* | F: CGWAG GGAAG CTGTT AAGT  T: AGCAC CACAA CGCGT GGA  R: TACCG TCGTC CACTC CTT | 343 |
| MMB282F  MMB749F  MMB832R | *Methanomicrobiales* | F: ATCGR TACGG GTTGT GGG  T: TYCGA CAGTG AGGRA CGAAA GCTG  R: CACCT AACGC RCATH GTTTA C | 506 |
| Msc380F  Msc492F  Msc828R | *Methanosarcinaceae* | F: GAAAC CGYGA TAAGG GGA  T: TTAGC AAGGG CCGGG CAA  R: TAGCG ARCAT CGTTT ACG | 408 |
| Mst702F  Mst753F  Mst862R | *Methanosaetaceae* | F: TAATC CTYGA RGGAC CACCA  T: ACGGC AAGGG ACGAA AGCTA GG  R: CCTAC GGCAC CRACM AC | 164 |

**_Adapted from Yu et al. (2005)_. F, T, and R indicate forward primer, TaqMan probe, and reverse primer, respectively.**
